# Protective effects of isoquercitrin on streptozotocin‐induced neurotoxicity

**DOI:** 10.1111/jcmm.15658

**Published:** 2020-08-01

**Authors:** Lei Chen, Peimin Feng, Anjiao Peng, Xiangmiao Qiu, Wanling Lai, Lin Zhang, Wanling Li

**Affiliations:** ^1^ Department of Neurology West China Hospital Sichuan University Chengdu China; ^2^ Department of Integrated Traditional and Western Medicine Hospital of Chengdu University of Traditional Chinese Medicine Chengdu China

**Keywords:** Alzheimer's disease, isoquercitrin, mitochondrial function, neurite outgrowth, streptozotocin

## Abstract

Alzheimer's disease (AD) is characterized by irreversible and progressive memory loss and has no effective treatment. Recently, many small molecule nature products have been identified with neuroprotective functions and shown beneficial effects to AD patients. In the current study, we thus performed a small scale screening to determine the protective effects of natural compounds on streptozotocin (STZ)‐induced neurotoxicity and Alzheimer's disease (AD). We found that a lead flavonoid compound, isoquercitrin (ISO) display the most effective anti‐cytotoxic activities via inhibiting STZ‐induced apoptosis, mitochondria dysfunction and oxidative stress. Treatment with ISO largely rescues STZ‐induced differentiation inhibition and enhances neurite outgrowth of Neuro2a (N2a) cells in vitro. Moreover, oral administration of ISO protects hippocampal neurons from STZ‐induced neurotoxicity and significantly improves the cognitive and behavioural impairment in STZ‐induced AD rats. In general, our screening identifies ISO as an effective therapeutic candidate against STZ‐induced neurotoxicity and AD‐like changes.

## 1 INTRODUCTION

Alzheimer's disease (AD) is a common progressive neurodegenerative disorder characterized by cognitive impairment and memory deterioration.^1^, ^2^ It is the most common cause of dementia worldwide, and there is a lack of drugs for the treatment of AD with satisfactory efficacy. The neurodegenerative process of the disease is characterized by unusual formation of amyloid‐β plaques and aggregation of tau proteins, accompanied by neuronal loss and synaptic damage, and there is increasing recognition of aetiologic factor in the disease.^1^, ^2^, ^3^, ^4^, ^5^, ^6^, ^7^, ^8^ The molecular pathogenesis of AD is very complex and still not fully understood. There is growing evidence that mitochondrial damage and oxidative stress are playing a critical role in the pathogenesis of AD, and the search for drugs that target mitochondria and antioxidants holds great promise. It has long been known that some naturally available small molecules have neuroprotection bioactivities to reduce neurodegeneration and to preventing the onset of neurodegenerative disease and to enhance cognitive functions.^9^, ^10^, ^11^ For example, it has been shown that Myricetin could suppress oxidative stress after ischaemic stroke and brain injury.^12^ It could also increase the number of hippocampal CA3 pyramidal neurons and improved learning and memory impairments in rodent model of Alzheimer's disease.^13^

Streptozotocin (STZ), a glucosamine‐nitrosourea compound derived from soil bacteria, was originally identified as an antibiotic and anticancer agent. It has been shown that administration of STZ in rat brain produces inflammation, oxidative stress and other AD‐like pathology, resulting in neuronal cell loss, cognitive dysfunctions and learning deficits.^14^, ^15^ In vitro, treatment with STZ induces cell death and expression of AD‐related markers in N2a cells.^16^ In the current study, we performed a small scale screening to identify natural compounds with putative anti‐AD activity using STZ‐treated N2a cells as a model. Together, 30 natural compounds representing eight major classes of natural products (ie alkaloid, anthraquinone, chromone, coumarin, flavonoid, phenolic acid, phenylpropanoid and terpenoid) with putative neuroprotection effects were included. N2a cells were treated with an LC_50_ dose of STZ (15 μmol/L) and two different concentrations (1 and 5 μmol/L) of the natural compounds, and cell viabilities were measured. Our screening identified isoquercitrin (ISO) as a lead flavonoid compound with the strongest anti‐cytotoxic activities.

We further examined the neuronal protective effects of ISO in both undifferentiated and differentiated N2a cells in vitro as well as in STZ‐induced AD rat brain in vivo. Although the mechanisms of AD pathogenesis are complex, there is accumulating evidence that oxidative stress and mitochondrial dysfunction are highly involved. Given the antioxidant features of flavonoid, we particularly focused on the antagonistic effects of STZ and ISO in regulating oxidative stress and mitochondrial function. Finally, we examined the effects of ISO in improving the cognitive and behavioural impairment induced by STZ in AD‐like rat model.

## 2 EXPERIMENTAL SECTION

### 2.1 Chemicals

Streptozotocin, caffiene, pseudoephedrine, emodin, sennoside, naringenin, chrysin, hesperidin, isoquercitrin, kaempferol, luteolin, myricetin, prunetin, quercetin, caffeic acid, chlorogenic acid, vanillin, allyl anthranilate, carvone, caryophyllene oxide and glycyrrhetinic acid were purchased from Sigma Aldrich (St. Louis, MO, USA). Epicatechin, rutin, vitexin and cynarin were purchased from Bedoukian Research (Danbury, CT, USA). Khellin, umbelliferone were obtained from Cayman Chemical Company (Ann Arbor, MI, USA). Gallic acid, cuminaldehyde, isoeugenol and thymol were purchased from Shenggong Research (Shanghai, China). Mcl‐1 inhibitor S63845 was obtained from APExBIO (Houston, TX, USA).

### 2.2 N2a cell culture and differentiation

Undifferentiated N2a cells were cultured in 24‐well plates with DMEM (Thermo Fisher, Chelmsford, MA, USA) containing penicillin/streptomycin (Thermo Fisher) and 10% heat‐inactivated FCS (Thermo Fisher). To induce differentiation, N2a cells were treated with DMEM containing 0.1% serum and 5 μmol/L RA (Sigma), together with the indicated natural compounds, for 24 hours. Differentiated N2a cells were stained with Neurite Outgrowth Staining Kit (Thermo Fisher) and imagined using an inverted microscope (Eclipse Ti‐E Inverted Microscope, Nikon, Japan). For each treatment group, about 200 randomly selected cells were counted to obtain the proportion of neurite‐bearing cells.

### 2.3 Western blot

Aliquots of 20 μg of proteins from total cell lysates were separated on 4%‐12% SDS–PAGE (Thermo Fisher) and transferred onto a polyvinylidene difluoride (PVDF) membrane (Millipore, Bedford, MA, USA). The membrane was blocked with 5% milk in PBST for 1 hour at room temperature and then subjected to immunoblotting using the following antibodies: anti‐Caspase‐3, anti‐Bax, anti‐Bcl2, anti‐VDAC and anti‐TIM23 antibodies (all from Cell signalling, Danvers, MA, USA); anti‐ATP5A and anti‐GAPDH antibodies (Abcam, Cambridge, UK) at 4°C overnight, followed by incubation with the corresponding secondary antibodies for 1 hour at room temperature. After 3X PBS wash, proteins were detected using enhanced chemiluminescence (ECL) detection kit (Amersham Biosciences, Piscataway, NJ, USA).

### 2.4 Real‐time PCR

Total RNA was isolated by Trizol reagent (Thermo Fisher) as per the manufacturer's instructions. 2 μg of total RNA was used to prepare cDNA using high capacity cDNA reverse transcription kit (Thermo Fisher). Real‐time PCR was performed using SYBR® Green Quantitative RT‐qPCR Kit (Sigma) based on the manufacturer's guidelines using the following primers: Tubb3 forward 5′‐ACACCTATTCAGGCCCGACAAC‐3′, reverse 5′‐CCGCACGACATCTAGGACTGAG‐3′; ChAT forward 5′‐GAGCCACCTGAGATGTTCATG‐3′, reverse 5′‐CAGCAGAACATCTCCATGGTC‐3′; ActB forward 5′‐TGAAGTGTGACGTTGACATCCG‐3′, reverse 5′‐GTACTCCTGCTTGCTGATCCAC‐3′. Real‐time PCR was performed on a ABI VIIA 7 Real‐Time PCR System (Applied Biosystems, Waltham, MA, USA).

### 2.5 Annexin V/PI staining and flow cytometry

Apoptosis was estimated by Annexin V‐FITC staining kit (Promega, Madison, WI, USA) as per the manufacturer's protocol. Cells were seeded in 12‐well plates for 24 hours and then exposed to different treatment. Following treatment, cells were suspended in binding buffer at a concentration of 1 × 10^6^ cells/mL and incubated with Annexin V‐FITC and propidium iodide (PI) for 10 minutes in the dark. Flow cytometry was performed using FACS Calibur and analysed by Cell Quest Pro software (BD Biosciences, Woburn, MA, USA).

### 2.6 Mitochondrial membrane potential and ATP production

Mitochondrial membrane potential was measured by Rhodamine 123 staining (Thermo Fisher) as per the manufacturer's instructions. Cells were incubated with 10 μg/mL Rhodamine 123 dye in the dark for 30 minutes at 37°C. After incubation, the cells were washed 3× with PBS and resuspended in 10^6^ cells/mL. Rhodamine 123 intensity was then analysed using FACS Calibur and analysed by Cell Quest Pro software (BD Biosciences, Woburn, MA, USA). ATP levels within the cell were measured by Luminescent ATP Detection Assay Kit (Abcam, Cambridge, UK).

### 2.7 ROS production

ROS‐specific staining was performed using DCFH‐DA (Molecular Probes, Eugene, OR, USA). N2a cells (1 × 10^5^) were seeded in cover slip loaded 6‐well plates. After different treatment, cells were incubated with 5 μmol/L DCFH‐DA for 20 minutes at 37°C. After washing 2× with PBS, cells were fixed with 4% formaldehyde and mounted on to microscope slides and visualized using the DP72 Olympus fluorescence microscope and cellSens software (Olympus, Waltham, MA, USA). Total DCFH‐DA fluorescence intensity was analysed by a spectrophotometer (BD Biosciences, Woburn, MA, USA).

### 2.8 MTT assay

Cell viability was estimated by using 3‐4,5‐dimethylthiazol‐2‐yl‐2,5‐diphenyltetrazolium bromide (MTT) dye (BD Biosciences) as per the manufacturer's protocol.

After treatment with STZ in combination with various natural compounds, cells were incubated with MTT dye for 2 hour at 37°C in the dark. The fluorescent intensity was measured by using spectrophotometer (BD Biosciences) at a wavelength of 550 nm with the reference wavelength of 630 nm.

### 2.9 Colony formation assay

N2a cells were digested by 0.25% trypsin and serially diluted into cell suspensions. About 10^4^ cells were incubated in 12‐well culture dishes for 6 days. After the formation of visible colonies, cells were rinsed in PBS and fixed in methanol for 15 minutes. Crystal violet (Tiancheng Biotech, Shanghai, China) was used to stain the cells for 10 minutes, followed by air drying.

### 2.10 Animals and treatment

Male Wistar rats (weighing 300‐350 g, 5 months of age) were obtained from Hunan Slack King of Laboratory Animal Co., Ltd. Rats were housed on a 12 hour light‐dark cycle at 25 ± 2°C, and in a relative humidity of 60%‐80%. Animals were fed on a diet of standard pellets and water and were allowed to acclimate to the housing conditions for 7 days prior to experimentation. STZ was dissolved in sterile 0.9% saline before the experiment and was administered into the lateral ventricle. Rats were randomly divided into three groups (n = 6 each). For the Saline + saline group, rats received intracerebroventricular injection of saline (10 μL for each lateral ventricle) on the first day, and saline (2 mL/kg) was orally given to the rats over a period of 10 days. For the STZ + saline group, rats received intracerebroventricular injection of STZ (3 mg/kg, 10 μL for each lateral ventricle) on the first day, and saline (2 mL/kg) was orally given to the rats over a period of 10 days. For the STZ + ISO group, rats received intracerebroventricular injection of STZ on the first day (day 0), and 15 mg/kg/day ISO dissolved in saline was orally given to rats over a period of 10 days (starting from day 0, 2 hours before STZ injection). The animal study followed ARRIVE (Animal Research: Reporting In Vivo Experiments) guidelines and was approved by the institutional animal care and use committee of Sichuan University (permission number: SC20180987Z). All efforts were made to minimize the pain and distress of experimental animals.

### 2.11 Behavioural test

A step‐through type passive avoidance test unit (O’Hara & Co., Tokyo, Japan) comprised of a bright and dark compartment was used for the shuttle box experiment. It consisted of two equally sized compartments, including a bright chamber and a dark chamber, which were separated by a guillotine door. Prior to the experiment, all animals were placed into the apparatus for 1 hour to allow habituation. The test consisted of two sessions. In the first session, each rat was initially placed in the bright chamber and as soon as the rat spontaneously entered the dark chamber, the guillotine door was closed, and an electric shock was given (1 Hz, 5 seconds, 1/5 MA). The second session was carried out 24 hours later. Each rat was again placed in the bright chamber for the retention test. The step‐through latency from the bright to dark compartment, which was recorded as the transfer latency time in seconds, and the total time spent in the dark chamber was recorded.

### 2.12 Statistical analysis

Statistical analysis was performed using the GraphPad Prism 7 software. Data were expressed as Mean ± SEM of all replicates. Student's t test was used for comparisons between the groups, and *P*‐value < 0.05 was considered significant.

## 3 RESULTS

### 3.1 Screening of natural compounds against STZ‐induced cytotoxicity

**FIGURE 1:**
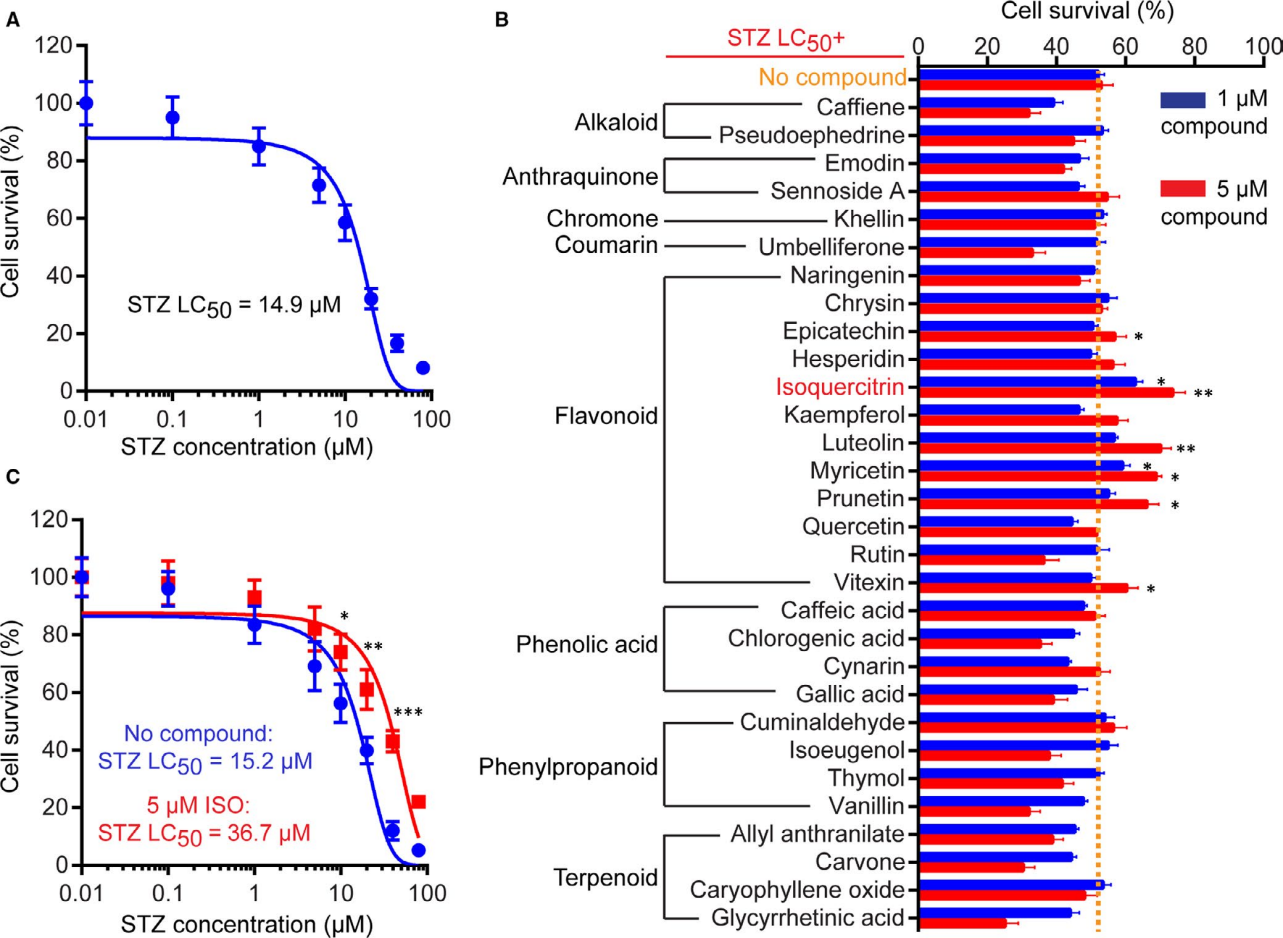
Screening of natural compounds against STZ‐induced cytotoxicity. A, Dose‐response curve of N2a cell viability upon treatment with different concentrations of STZ. The lethal concentration 50 (LC_50_) of STZ for the N2a cells used in this study is shown. B, Overview of primary screening data. N2a cells were treated with LC_50_ of STZ alone (no treatment) or in combination with individual natural compounds at two concentrations (1 and 5 μmol/L). Cell viability was analysed by MTT assay and normalized to DMSO‐treated control cells. Data represent mean ± SD of three biological replicates. **P < 0.01; *P < 0.05. C, Dose‐response curve of N2a cell viability upon treatment with different concentrations of STZ alone or in the present of 5 μmol/L ISO. The lethal concentration 50 (LC_50_) of STZ under the two conditions is shown

Intracerebroventricular injection of STZ in rat brain causes neurotoxicity and prolonged impairment of brain function, leading to cognitive dysfunction and other AD‐like changes.^2^, ^14^, ^15^ Our prime objective was to screen for novel natural compounds with protective effects on STZ‐induced neuronal cytotoxicity. To this end, we used STZ‐treated N2a cell as an in vitro experimental model for the screening. N2a is a mouse neuroblastoma cell line that could be differentiated into cells with many properties of neurons in vitro. We first treated N2a cells with different concentrations of STZ (0.01‐100 μmol/L) for 48 hours to determine the LC_50_ (14.9 μmol/L, Figure 1A). N2a cells were then treated with an LC_50_ dose of STZ (15 μmol/L) in combination with two different concentrations (1 and 5 μmol/L) of the 30 natural compounds belonging to 8 major classes (ie alkaloid, anthraquinone, chromone, coumarin, flavonoid, phenolic acid, phenylpropanoid and terpenoid) for 48 hours (Figure 1B and Table S1). Based on cell viability assays, our results showed that treatment with the higher dose (5 μmol/L) of several flavonoids, including isoquercitrin, luteolin, myricetin and prunetin, could provide significant protection against STZ‐induced cytotoxicity (Figure 1B). The lead compound isoquercitrin (ISO), also known as quercetin‐3‐glucoside, is a glucose‐bound derivative of quercetin with many biological activities, including anti‐inflammatory, anti‐oxidative and anti‐apoptotic effects. Co‐treatment with 5 μmol/L ISO significantly enhanced cell tolerance to different concentrations of STZ, increasing the LC_50_ of STZ from 15.2 to 36.7 μmol/L (Figure 1C). Together, our screening identified ISO as a lead flavonoid compound with anti‐cytotoxic activities against STZ, and we then investigated the anti‐oxidative and neuroprotective effects of ISO in different cellular and animal models.

### 3.2 Isoquercitrin protects against STZ‐induced cell death

**FIGURE 2:**
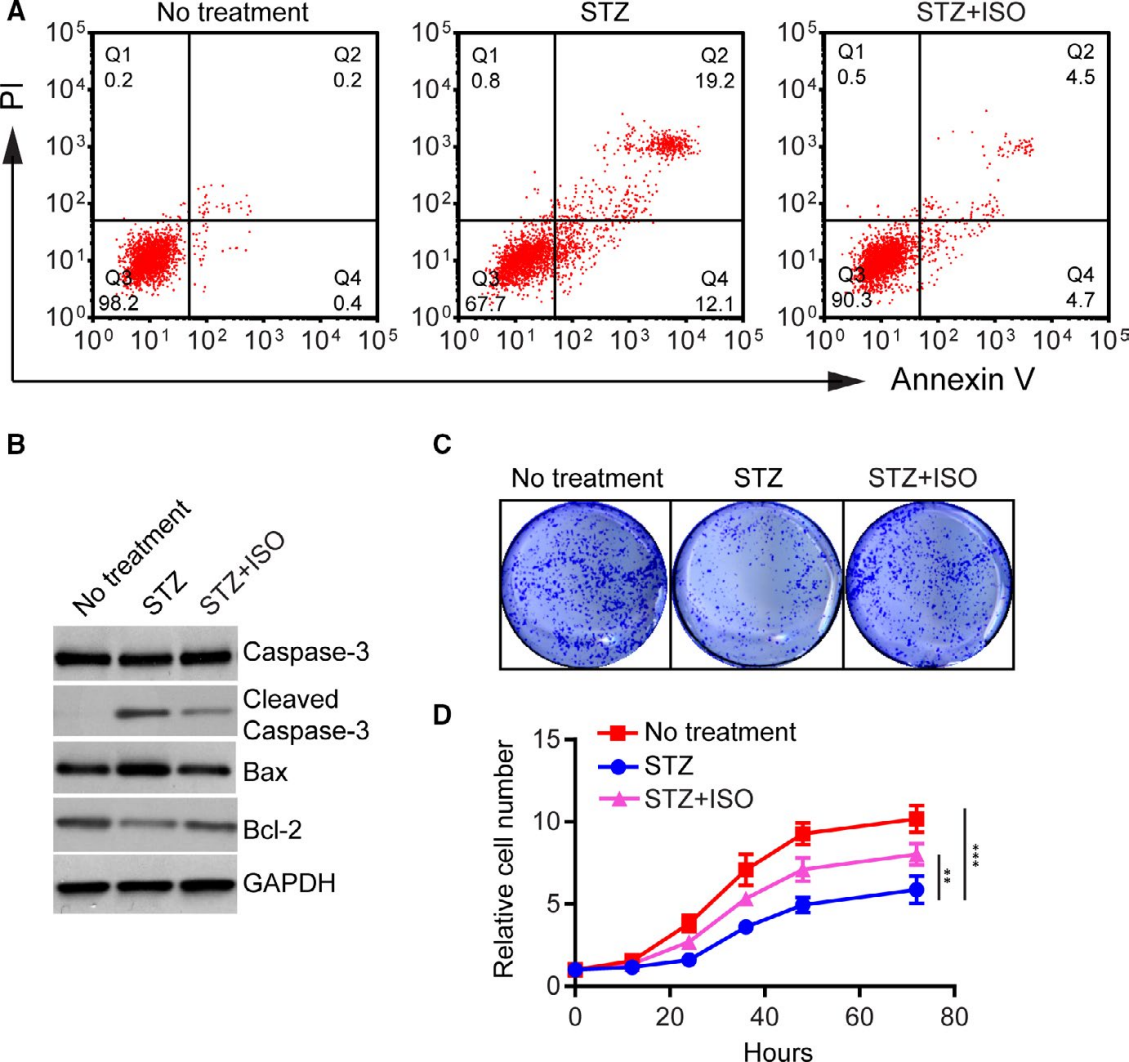
Isoquercitrin protects against STZ‐induced cell death. A, Flow cytometry analysis of cells with no treatment, treated with STZ (15 μmol/L) alone or in combination with ISO (5 μmol/L) for 48 h. Cells were stained with Annexin V‐FITC and propidium iodide (PI) dyes. The cells in Q2 and Q4 regions were considered apoptotic cells. B, Western blot of apoptosis‐related protein expression in different groups. C, Clonal formation assay of cells in different groups. About 10^4^ cells were incubated in 12‐well culture dishes for 6 d. After the formation of visible colonies, cells were fixed and stained with crystal violet. D, Growth curves of cells in different groups within 72 h. Data represent mean ± SD of three biological replicates. **P < 0.01; ***P < 0.001

We examined the anti‐apoptotic effect of ISO against STZ‐induced cell death. Cells with no treatment, treated with STZ (15 μmol/L) alone or in combination with ISO (5 μmol/L) for 48 hours were stained with Annexin V‐FITC and propidium iodide (PI) dyes and subjected to flow cytometry analysis (Figure 2B). Consistent with its cytotoxic effect, STZ treatment caused dramatic increase in the number of apoptotic cells compared with no treatment (31.3% vs 0.6%, Figure 2B). Combined treatment with ISO, however, largely reduced the number of apoptotic cells induced by STZ (9.2% vs 31.3%). As shown in Figure 2B, Western blot analysis revealed that ISO treatment significantly increased cleavage of Caspase‐3, up‐regulated expression of pro‐apoptotic protein Bax and down‐regulated expression of anti‐apoptotic protein Bcl‐2, indicating apoptotic cell death. In contrast, co‐treatment with ISO led to potent decrease of Caspase‐3 cleavage and enhancement in Bcl‐2 expression, supporting an anti‐apoptotic effect of ISO. Consistent with their higher apoptotic index, STZ‐treated N2a cells showed significantly reduced colony formation and cell proliferation compared to cells with no treatment (Figure 2C‐D). Co‐treated with ISO, however, could largely rescue the proliferation defect induced by STZ (Figure 2C‐D). Together, these data suggest that ISO could efficiently protect N2a cells against STZ‐induced cell death and proliferation arrest.

### 3.3 Isoquercitrin protects against STZ‐induced mitochondrial dysfunction and oxidative stress

**FIGURE 3:**
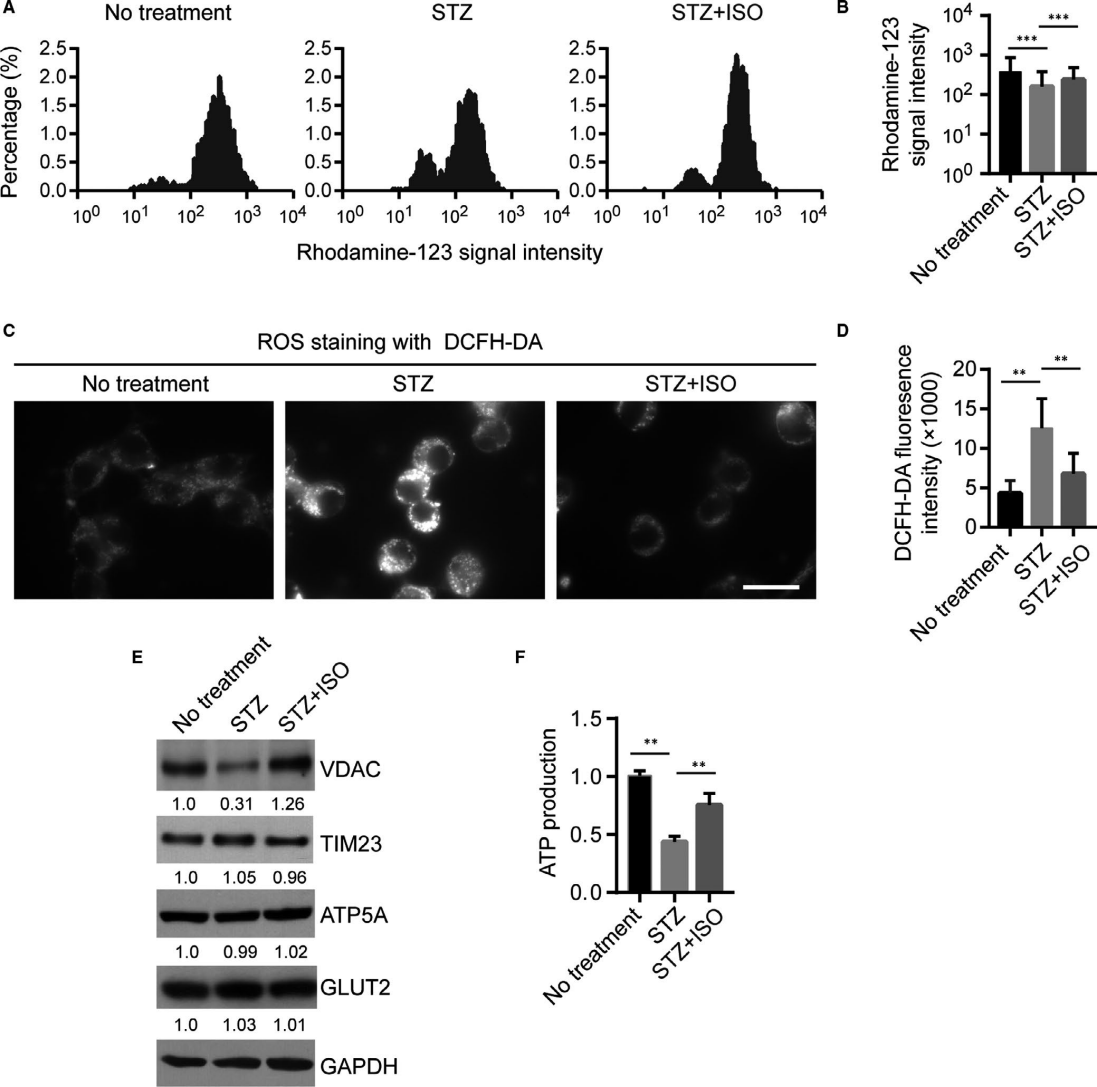
Isoquercitrin protects against STZ‐induced mitochondrial dysfunction and oxidative stress. A, Histogram plotting the Rhodamine‐123 signal intensities in cells upon different treatment identified by flow cytometry analysis. *X*‐axis is log transformed. B, Statistics of Rhodamine‐123 signal intensities identified in (A). *Y*‐axis is log transformed. ***P < 0.001. C, ROS staining with DCFH‐DA in different groups of cells. Scale bar: 15 μm. D, Statistics of DCFH‐DA signal intensities identified in (C). **P < 0.01. E, Western blot of expression of different mitochondrial proteins in different groups, including the outer mitochondrial membrane proteins VDAC, the inner mitochondrial membrane protein TIM23, mitochondrial matrix protein ATP5A and the glucose transporter GLUT2. F, ATP production in different groups measured by Luminescent ATP Detection Assay

As regulators of both energy metabolism and cell death pathways, mitochondria are playing a critical role in neuronal cell survival and function. Previous studies suggested that mitochondrial dysfunction and oxidative stress are tightly linked to the early pathogenesis of AD.^5^, ^7^, ^8^, ^17^ It has been shown that mitochondrial membrane potential and enzyme activities were disrupted in STZ‐treated cells, resulting in reactive oxygen species (ROS) formation and inhibition of ATP production.^17^, ^18^ Consistent with these results, using Rhodamine‐123 staining, we showed that mitochondrial membrane potential was significantly reduced in N2a cells treated with STZ (15 μmol/L, 48 hours) compared to cells with no treatment (Figure 3A‐B). Of note, addition of ISO (5 μmol/L) significantly attenuated the mitochondrial membrane potential loss induced by STZ (Figure 3A‐B), supporting a direct role of ISO in enhancing mitochondrial function. Furthermore, STZ‐induced oxidative stress and ROS production were detected by DCFH‐DA staining (Figure 3C‐D). Treatment with ISO markedly prevented the increase in mitochondrial ROS production by STZ (Figure 3C‐D).

Using Western blot, we then examined protein expression of several key mitochondrial proteins, including the outer mitochondrial membrane proteins VDAC, the inner mitochondrial membrane protein TIM23 and ATP5A for matrix. The expression levels of glucose transporter protein GLUT2 have also been examined. Our results showed that STZ treatment resulted in significant reduction in VDAC protein expression, but had no influence on TIM23, ATP5A or GLUT2 expression (Figure 3E). Notably, co‐treatment with ISO could largely enhance expression of VDAC (Figure 3E). Given the critical roles of VDAC in regulating Ca^2+^ homeostasis, apoptosis and metabolic activities, it is possible that the protective effects of ISO on STZ‐induced mitochondrial dysfunction is at least partially via restoring VDAC expression. Moreover, STZ treatment resulted in potent reduction in ATP production in N2A cells (Figure 3F), and this could be largely rescued by combined treatment with ISO (Figure 3F). Together, these results support a model that ISO protect STZ‐induced cell death through restoring mitochondria function.

### 3.4 Isoquercitrin attenuates STZ‐induced N2a differentiation defects

**FIGURE 4:**
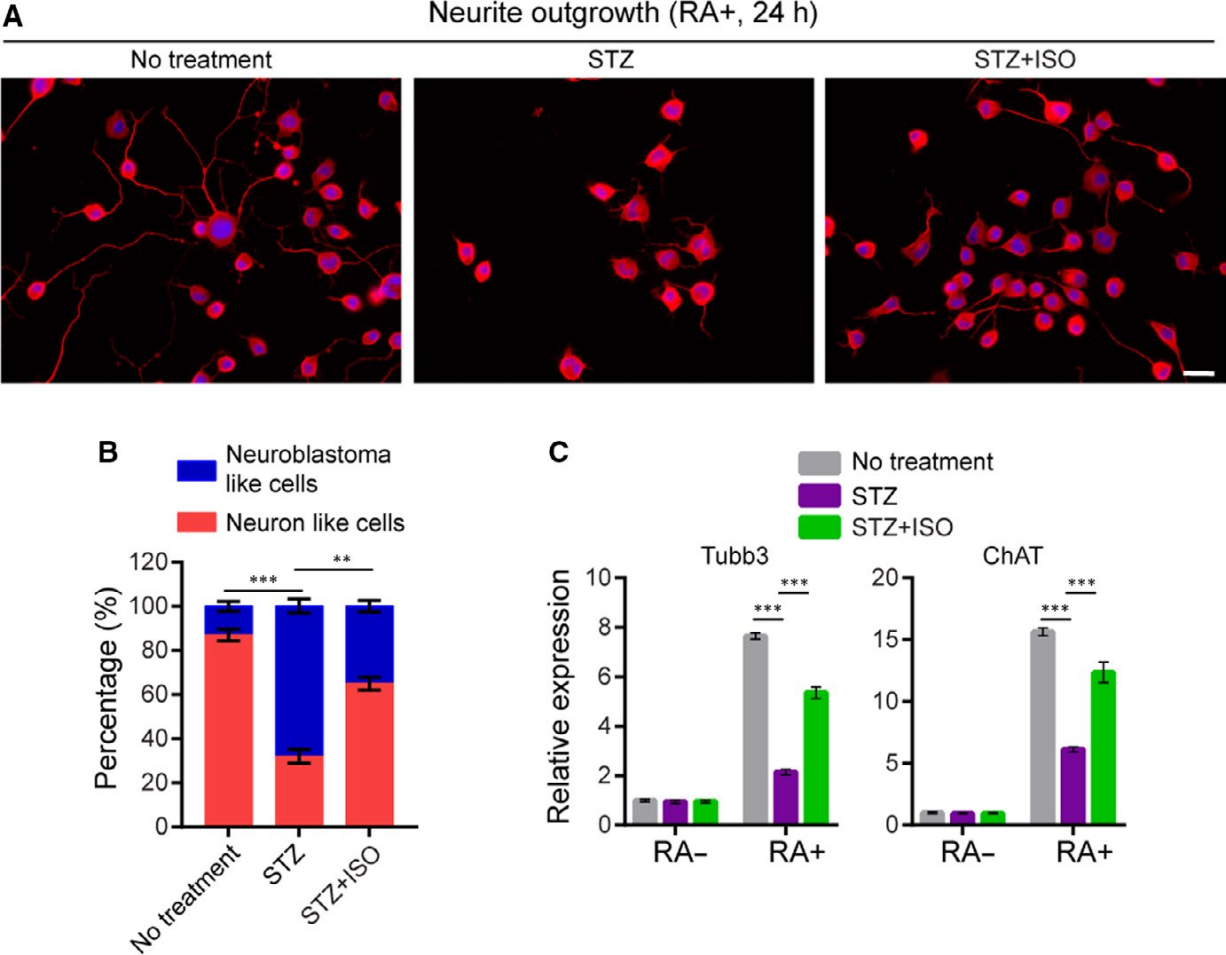
Isoquercitrin attenuates STZ‐induced N2a differentiation defects. A, Representative data of RA‐induced differentiation and neurite outgrowth in N2a cells upon different treatment. N2a cells were treated with DMEM containing 0.1% serum and 5 μmol/L RA, together with the indicated treatment for 24 h. Cells were exposed to 20 μmol/L retinoic acid in the absence of serum for 24 h. Cell membrane (red) was staining with Neurite Outgrowth Staining Kit and nucleus was stained with DAPI (blue). Scale bar: 15 μm. B, Quantitative analysis of the proportion of differentiated N2a cells upon different treatment. Cells with clear outgrowth of neurites were defined as neuron‐like cells. Data represent mean ± SD of three biological replicates. About 200 (×3) cells were counted for each sample. C, Real‐time PCR analysis of ChAT and Tubb3 expression in undifferentiated and differentiated cells upon different treatment. Data represent mean ± SD of three biological replicates. ***P < 0.001; **P < 0.01

To determine the activity of ISO to promote the recovery of neuronal functions, we examined neurite outgrowth in N2a cells, a well‐established model system of neuronal differentiation in vitro. Upon serum starvation and treatment with retinoic acid (RA), N2a cells undergo a series of physiological changes culminating in a phenotype resembling that of cholinergic neurons. Using this system, N2a cells pre‐treated with STZ alone (15 μmol/L), in combination with ISO (5 μmol/L), or with no treatment for 48 hours, were differentiated for another 24 hours and the proportion of neuron‐like cells with robust outgrowth of neurites was calculated. As shown in Figure 4A no treatment group, the typical phenotype of neurons differentiated from N2a cells is shown. Treatment with STZ, however, largely inhibited N2a cell differentiation, resulting in reduced proportion of neuron‐like cells and shortened neurites (Figure 4A‐B). Treatment with ISO largely attenuated STZ‐induced N2a differentiation defects and significantly enhanced the number of neuron‐like cells (Figure 4A‐B). Notably, ISO treatment alone could not increase N2a cell differentiation compared to control (Figure S1A‐B), suggesting that ISO largely exert its function through remedy of STZ‐induced cellular dysfunction.

Furthermore, we examined expression of neuronal cell markers, Tubb3 and ChAT, in different groups of cells using real‐time PCR. As expected, in no treatment group, neuronal cell differentiation is highly correlated with elevated expression of the neuronal markers (Figure 4C). In STZ‐treated cells, however, our results showed that expression of both ChAT and Tubb3 were significantly reduced compared with no treatment group (Figure 4C), consistent with defective neuronal differentiation. ISO treatment largely restored ChAT and Tubb3 expression in differentiated cells treated with STZ (Figure 4C). Together, these results revealed the neurite outgrowth‐promoting activity of ISO, independent of its cell survival‐promoting activity.

### 3.5 Neuroprotective effects of isoquercitrin in STZ‐treated rat brain

It has been well‐established that intracerebroventricular injection of STZ leads to AD‐like changes such as prolonged neuronal cell impairment and cognitive dysfunction.^13^, ^14^, ^16^ Using this model, we thus further examined the protective effects of ISO on STZ‐induced neurotoxicity in rat brain. Rats received one‐time intracerebroventricular injection (10 μL for each lateral ventricle) of saline or STZ (3 mg/kg dissolved in saline) and continued with oral administration of saline (2 mL/kg/d) or ISO (15 mg/kg/d)^19^, ^20^ dissolved in saline over a period of 10 days (starting from day 0). After that, brain sections were stained with haematoxylin‐eosin and the passive avoidance test was performed to evaluate learning and memory in the rats.

**FIGURE 5:**
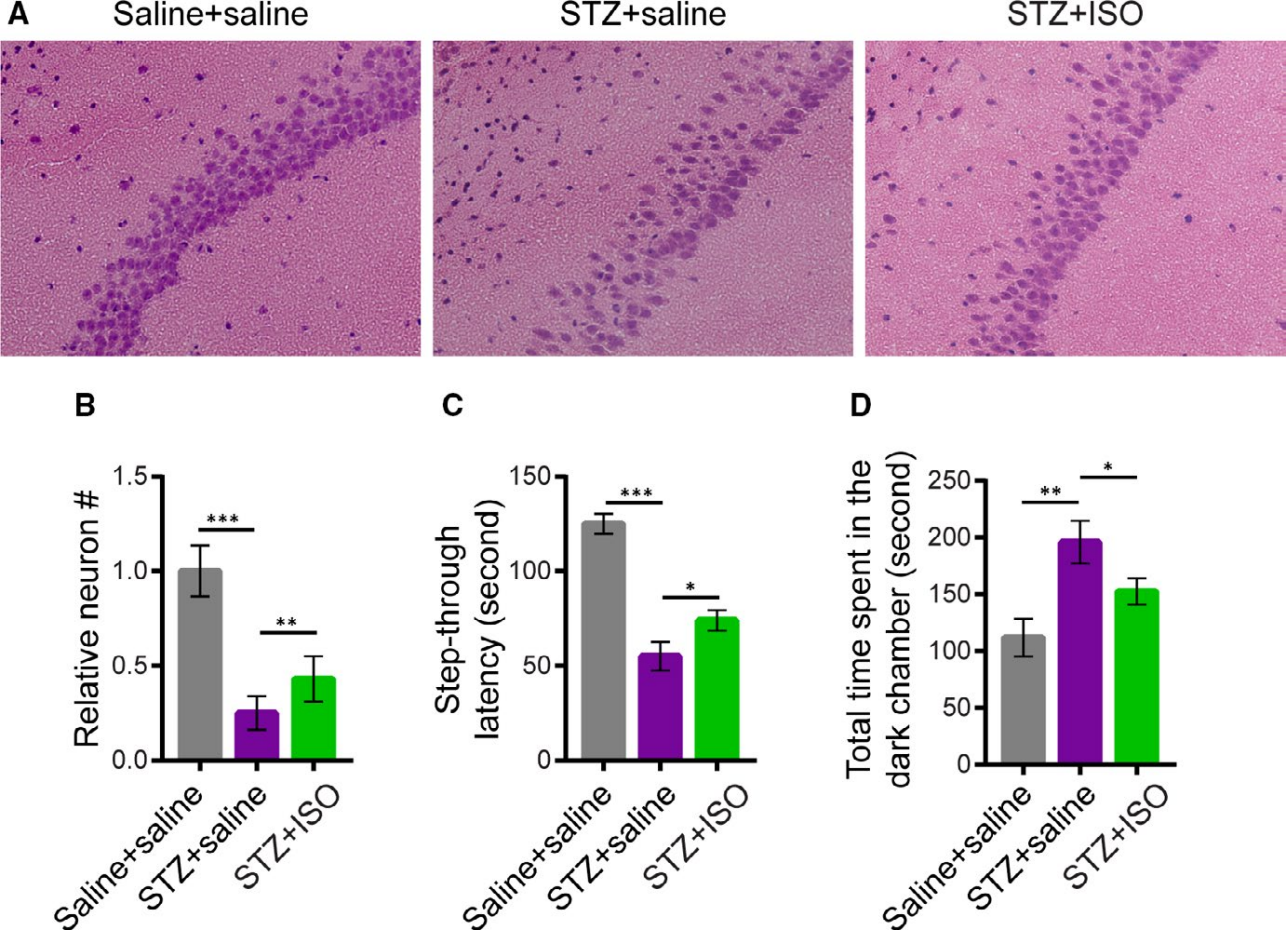
Neuroprotective effects of isoquercitrin in STZ‐treated rat brain. A, Haematoxylin‐eosin staining of hippocampal pyramidal CA3 neurons of rats upon different treatment. Rats were randomly divided into three groups (n = 6 each). For the Saline + saline group, rats received intracerebroventricular injection of saline (10 μL for each lateral ventricle) on the first day and continued with oral administration of saline (2 mL/kg) for 10 d. For the STZ + saline group, rats received intracerebroventricular injection of STZ (3 mg/kg, 10 μL for each lateral ventricle) on the first day and oral administration of saline for 10 d. For the STZ + ISO group, rats received intracerebroventricular injection of STZ on the first day (day 0) and oral administration of ISO dissolved in saline (15 mg/kg/d) for 10 d. B, The number of neurons in the field were counted and normalized by the saline control. Data represent mean ± SD of three biological replicates. C, Step‐through latency and D, total time spent in the dark room of passive avoidance apparatus for different groups of rats as described in (A). ***P < 0.001; **P < 0.01; *P < 0.05

Consistent with previous findings, injection of STZ in rat brains resulted in severe hippocampal CA3 pyramidal neuronal damage characterized by significant neural loss and irregularities in the layer (Figure 5A). In contrast, injection with saline did not cause any severe neuronal damage in rat brain (Figure 5A). Statistical analysis revealed that the number of neurons in the hippocampal CA3 pyramidal layer was significantly reduced in the STZ+ saline group vs the Saline + saline group (*P* < 0.01, Figure 5B). In line with its neuroprotective effects in vitro, oral administration of ISO (STZ + ISO group) could partially attenuated STZ‐induced neuron loss and irregular layer organization in the hippocampus (Figure 5A‐B), supporting its function in vivo.

STZ‐induced hippocampal defects result in prolonged learning and memory impairment.^1^, ^12^, ^13^ To investigate the positive influences of ISO on cognitive functions of STZ‐treated rats, the shuttle box‐based passive avoidance test was performed (see methods). The test is used to assess memory function about a specific hazardous environmental context, which the animal learns to avoid. Our results showed that the step‐through latency to enter the dark room of passive avoidance apparatus was significantly shorter in the STZ + saline group (Figure 5C), and the total time spent in the dark room was significantly longer (Figure 5D) compared with the saline group, indicative of compromised cognitive functions of STZ‐treated rats. In contrast, however, oral administration of ISO could partially enhance learning and memory in STZ‐treated rats, resulting in significantly elongated step‐through latency and shortened time spent in the dark room of passive avoidance apparatus compared to the STZ + saline group (Figure 5C‐D). These results are consistent with the neuroprotective effects of ISO in vitro and in vivo, suggesting that the use of natural products against STZ‐induced neurodegeneration might therefore provide new insights into drug development for AD treatment.

## 4 DISCUSSION

Natural compounds are often appreciated for providing various beneficiary health effects with low systemic toxicity. An abundancy of natural resources has shown promising anti‐neurotoxicity and neurogenesis stimulating activities.^11^, ^21^ In the current study, we systematically evaluated the protective effects of numerous natural compounds against STZ‐induced cytotoxicity. Treatment with STZ induces oxidative stress and AD‐like changes in the rat brain, providing a model to explore the underlying pathophysiological mechanisms. Our screening identified ISO as a lead flavonoid compound with the best anti‐cytotoxic activities. Using different cellular and animal models, we further showed that ISO could largely rescue STZ‐Induced apoptosis, mitochondria dysfunction and oxidative stress. It also has great beneficial effects on neuronal functions such that it enhances differentiation and neurite outgrowth of N2a cells and protects hippocampal neurons from STZ‐induced neurotoxicity. Moreover, our results showed that ISO could significantly improve the cognitive and behavioural impairment in STZ‐treated rats.

Oxidative stress and neuro‐inflammation of the brain have been implicated as leading risk factors of AD. Flavonoids exist in many vegetables and fruits, and their most important characteristics involve antioxidant activity and the ability to suppress reactive oxygen species formation.^9^, ^22^ Given these properties, dietary flavonoid could be used as an effective therapeutic candidate against AD. Our results are consistent with prior findings regarding the antioxidant and neuroprotective effects of ISO.^23^, ^24^, ^25^, ^26^ Its effects to promote neurite growth are also supported by prior findings in mouse NG108‐15 cells.^27^ Although ISO can induce chromosomal aberrations in CHO‐WBL cells, however, there is no evidence that ISO induces substantial genotoxicity in vivo, supporting its safety in food and beverage products.^28^

Consistent with this notion, in addition to ISO, our screening also identified several flavonoids, including luteolin, myricetin and prunetin, that also provided significant protection against STZ‐induced cytotoxicity. Indeed, accumulating studies provide evidence supporting the neuroprotective activities of flavonoids and combining these properties with their availability in human diets thus provide important insight into dietary therapy against the disease. For example, it has been shown that ISO inhibits H_2_O_2_‐Induced apoptosis of EA.hy926 cells via activation of the PI3K/Akt/GSK3 signalling pathway.^29^ Moreover, prior studies showed that myricetin ameliorates brain injury and neurological deficits via Nrf2 activation and restoring mitochondrial function in stroke rat model.^12^ It has also been shown that Myricetin protects provides neuroprotection and improves learning and memory impairments in AD rat model.^13^ Notably, recent studies have identified innumerable compounds from natural sources which could control and conserve neuronal survival, differentiation and long‐term potentiation in memory. Combination of different compounds with plentiful active constituents might thus be particularly useful in terms of neuroprotection and neuronal functions improvement. A clear understanding of the mechanisms of action of these natural compounds, either as antioxidants or modulators of cell signalling pathways, will provide novel insights into drug development for neurodegeneration diseases.

The metabolism and pharmacokinetics of ISO have been well‐established. Its biotransformation involves deglycosylation followed by formation of conjugated and methylated derivatives of quercetin. It has been shown that ISO can be metabolized into Quercetin and quercetin‐3‐O‐β‐D‐glucuronide, both have good anti‐oxidative and anti‐inflammatory functions.^30^ Otherwise, it is degraded to phenolic acids and carbon dioxide. Previous studies of the pharmacokinetics of dietary flavonoids in humans revealed an intestinal absorption ranging from 0% to 60% and elimination half‐lives ranging from 2 to 28 hours,^31^ and the proper daily intake of (95%) ISO was estimated to be 5 mg/kg/d.

In conclusion, using both in vitro and in vivo models, our study identified the neuroprotective activity of ISO against toxicity induced by STZ in a AD rat model. The great safety, affordability and beneficial pharmacological activities of ISO thus make it a good supportive treatment for patients suffering from AD.

## CONFLICT OF INTEREST

The authors confirm that there are no conflicts of interest.

## AUTHOR CONTRIBUTION

**Lei Chen:** Conceptualization (lead); Data curation (lead). **Peimin Feng:** Data curation (equal); Formal analysis (equal); Investigation (equal). **Anjiao Peng:** Resources (equal); Software (equal). **Xiangmiao Qiu:** Data curation (supporting); Investigation (supporting). **Wanling Lai:** Data curation (supporting). **Lin Zhang:** Formal analysis (lead). **Wanling Li:** Data curation (supporting); Resources (supporting).

## Supporting information

Fig S1

Table S1
