# Supplementary material for: Protective effects of isoquercitrin on streptozotocin‐induced neurotoxicity
Source: J Cell Mol Med. 2020 Aug 1;24(18):10458–67. doi: 10.1111/jcmm.15658 (PMC7521287; doi:10.1111/jcmm.15658)
Supplement: Supplementary file 1 — Fig S1 [file JCMM-24-10458-s001.pdf]

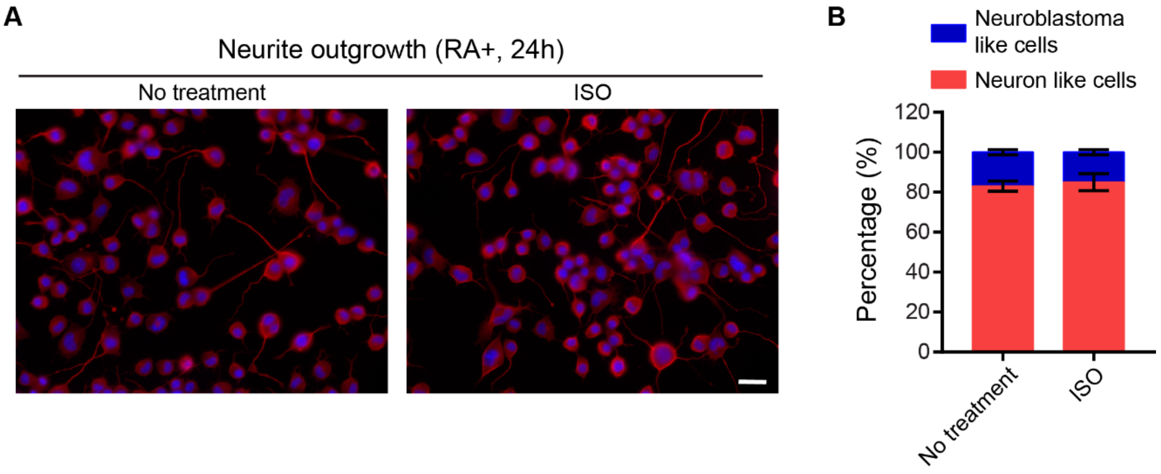

Supplementary Fig.1. Isoquercitrin has no direct influence on N2a differentiation. (A) Representative data of RA-induced differentiation and neurite outgrowth in N2a cells upon different treatment. N2a cells were treated with DMEM containing 0.1% serum and 5 μM RA, with or without 5 μM ISO for 24h. Cells were exposed to 20 μM retinoic acid in the absence of serum for 24 hours. Cell membrane (red) was staining with Neurite Outgrowth Staining Kit and nucleus was stained with DAPI (blue). Scale bar: 15 μm. (B) Quantitative analysis of the proportion of differentiated N2a cells. Cells with clear outgrowth of neurites were defined as neuron like cells. Data represent mean±SD of three biological replicates. About 200 (×3) cells were counted for each sample.
